# Psychometric Properties of the Indebtedness Scale (IS-R) in Spanish University Students

**DOI:** 10.3389/fpsyg.2019.01094

**Published:** 2019-07-17

**Authors:** Gloria Bernabé-Valero, Carmen Moret-Tatay, Isabel Iborra-Marmolejo, José Salvador Blasco-Magraner

**Affiliations:** ^1^Departamento de Ciencias de la Ocupación, Logopedia, Psicología Evolutiva y de la Educación, Universidad Católica de Valencia, San Vicente Mártir, Valencia, Spain; ^2^Departamento de Metodología, Psicología Básica y Psicología Social, Universidad Católica de Valencia, San Vicente Mártir, Valencia, Spain; ^3^Departamento de Didáctica de la Expresión Musical, Plástica y Corporal, Universidad de Valencia, Valencia, Spain

**Keywords:** indebtedness, gratitude, psychometric properties, scale, confirmatory analysis, positive psychology

## Abstract

The importance of trait indebtedness in the context of the study of gratitude has been growing in recent years, since both constructs form complex dynamics in response to the reception of a gift. In this work, the disposition to feel indebtedness is studied, through analysis of convergence and divergence, exploratory, and confirmatory analysis in the Spanish population, with the most used measurement instrument, i.e. The Revised Indebtedness Scale, IS-R. This scale depicted a four factor solution interrelated with a high consistency of content, which allows their labeling and describing. To do this, two samples of university students were selected; one of the sample sizes had 229 participants and the other 200 participants. Results also indicated good internal consistency described as follows: Debt for material aspects (α = 0.81), Self-sufficiency and discomfort in receiving help (α = 0.82), Moral self-demand in the reception of help (α = 0.83) and Debt in the receipt of gifts (α = 0.75). Furthermore, it was suggested that the relationships between gratitude and indebtedness are complex: on the one hand, all the indebtedness factors are inversely correlated with gratitude, such as the GQ5, although only Self-sufficiency and discomfort in receiving help and Debt in the receipt of gifts are such to a significant extent. However, the Interpersonal Gratitude scale of the G-20 depicted low correlations only with the Self-sufficiency and discomfort. The results are discussed in relation to the need for conceptual definition of the constructs in Positive Psychology.

## Introduction

Positive Psychology, already established as a fruitful field since the beginning of this century, has advanced at a theoretical and empirical level in its most relevant constructs, such as well-being, gratitude, happiness and virtues, among others (Setti et al., 2018). These advances have led to the identification of obstacles that may impede or inhibit the development of some positive experiences. For example, Solom et al. (2016) found that variables such as cynicism, materialism, envy, narcissism and indebtedness were inversely related to gratitude.

The importance of gratitude in the current field of psychology is indisputable. In this way, they have considered that enhancing gratitude is a way of nurturing happiness (Watkins et al., 2019). For that reason, we are interested in one of the variables that has been most related to the inhibition of the emergence of interpersonal gratitude, the construct of indebtedness (Watkins, 2014).

Although some theorizations have attempted to equate gratitude with indebtedness, arguing that returning a benefactor a favor could occur due to both indebtedness and gratitude, the researchers have attempted to define the differences between both concepts.

Firstly, it has been argued that indebtedness is accompanied by negative emotions, linked to discomfort (Greenberg, 1980), whereas gratitude is an emotion with positive valence (Mayer et al., 1991; Lazarus and Lazarus, 1994). Based on her theory of expansion and construction of positive emotions, Fredrickson (2004) proposes that when experiencing gratitude as a pleasant emotion and debt as aversive, only gratitude could lead to a comprehensive and creative thought on how to return a favor. Secondly, indebtedness is associated with motives of avoidance, while gratitude is associated with rapprochement and prosocial motivations (Gray et al., 2001). Therefore, when it is understood that the intentions of the benefactor are benevolent, a greater response of gratitude occurs (Tsang, 2006a), whereas the reasons or expectations of reciprocity of the benefactor would lead to an indebtedness response (Watkins et al., 2006). Thirdly, indebtedness arises from the reciprocity rule, while gratitude can go above and beyond the “eye for an eye” mentality (Greenberg, 1980). Hence, gratitude might imply the perception and recognition of a gift, which is conceived as valuable, for which a positive emotion of connection with the donor is experienced, whilst indebtedness would imply a state of obligation to return a benefit to another, with an emotional experience of arousal and discomfort (Greenberg, 1980). Goei and Boster (2005) studied the reciprocal behavior by making conceptual distinctions between obligation and gratitude, and through two experiments demonstrated that obligation and gratitude can be empirically distinguished.

Ting (2017) agrees that debt and gratitude are two different aspects, since the feelings of gratitude of the beneficiary can easily occur when the benefactor has no purpose, has low expectations of return, his help is disinterested, sincere, and voluntary; while indebtedness can be produced by the interpretation of contrary attitudes in the benefactor. In addition, this author differentiates between internal and external indebtedness. The first is generated after the beneficiary feels grateful, while the external (conventional) debt refers to indebtedness produced by external factors, of which they are unable to evoke feelings of gratitude in the beneficiary. This distinction sheds light on the fact that on some occasions, both the emotion of gratitude and the debt may emerge. Furthermore, the author highlights that, in situations of internal indebtedness, first the individual feels gratitude and then, he himself, feels debt and motivation to return the favor; however, in external indebtedness, gratitude would not appear.

Along the same lines, other authors such as Peng et al. (2018) and Bock et al. (2016) claimed that both variables should be studied together in order to delimit their complex dynamics.

Cross-cultural comparative studies have attempted to delimit the gratitude and debt component according to the culture of origin. For example, a study with 115 female Japanese university students confirmed the distinct meanings of gratitude, sumanai, and indebtedness in terms of their dissimilar correlations with other feelings. The results also revealed that when the benefactor’s expectation for repayment was manipulated, gratitude and sumanai, but not indebtedness, decreased with increasing benefactor expectations (Washizu and Naito, 2015). Differences have also been found in the use of expressions that involve gratitude, debt and other emotions involved in the expression “I’m sorry” (Kotani, 2002). Another study (Naito et al., 2005) compared Japanese and Thai students, finding positive feelings correlated with facial and verbal expressions of gratitude and increased prosocial motivation, whereas the feelings of indebtedness were positively related to increased prosocial motivation only in Japanese male students. Recent studies (Oishi et al., 2019) have indicated that gratitude evoked indebtedness among Korean students, but not among American students. To our knowledge there are no comparative studies with Spanish or Spanish-speaking populations. It could be considered interesting to know the distinctive cultural elements of debt and gratitude in the Spanish population, as this would allow us to better understand the complex functioning of these variables and thus to distinguish what can be extrapolated from the studies carried out with other cultural contexts. To be able to advance in this area, measurement instruments adapted to each population are required.

Various procedures have been used to measure gratitude and debt. Both have been conceptualized and measured as states (emotions) and as traits. From the perspective of debt as emotion, this has been measured in relation to the response evoked before a gift (Tsang, 2007; Naito and Sakata, 2010; Watkins et al., 2017). Some authors have measured it by means of an item before receiving a gift (Algoe et al., 2010; Gordon et al., 2012). For example, Algoe et al. (2010) in the context of the partner relationship were exposing the participants to these questions: “People feel many different things as a result of others’ actions on any given day or at any given time. Gratitude was assessed with three items, thankfulness, appreciation, and gratitude, and indebtedness was measured with a single item. Another measure widely used is the one created by Tsang (2006b) to assess current gratitude and indebtedness regarding the receipt of a specific benefit. For this purpose, participants rated emotion words (grateful, indebted, thankful, obligated, and appreciative) on a scale of 1 to 7 regarding the recalled favor or gift (see, for example, Mathews and Green, 2010; Mathews and Shook, 2013). Watkins et al. (2006), after the presentation of vignette with different situations, asked participants to determine if they in fact would experience the emotion (responding in a yes/no format), and if they indicated “yes” they were to rate the extent of their emotional response ranging from 1 (“mild”) to 4 (“extreme”). In the case of indebtedness, in case the term was not familiar to some of participants, they included a brief definition in parentheses after the term (“feeling obligated to repay”). What all these methods have in common is trying to elicit the emotion of debt (among others) and asking the participants to score them.

Debt as a trait has also been the subject of studies (Watkins et al., 2005, 2017; Mathews and Green, 2010; Mathews and Shook, 2013; Washizu and Naito, 2015). The measure that has been used the most to quantify the experience of indebtedness is the Revised Indebtedness Scale (IS-R, Elster et al., 2005; Van Gelder et al., 2007). This scale, based on the original Greenberg (1980) scale, was designed to measure the tendency of individuals to respond to benefits received with feelings of indebtedness (feeling obligated to pay others). This measure showed a good internal consistency, and in addition, it correlated negatively with the gratitude trait. To our knowledge, there are no previous studies that have used the indebtedness trait measure with the Spanish population and with the Spanish language. So our main aims will be to analyze the psychometric properties of the Indebtedness Scale (IS-R, Elster et al., 2005; Van Gelder et al., 2007) in the Spanish population. For this reason, exploratory (objective I) and confirmatory (II) studies will be carried out to see if the scale can be divided into factors and if they have content coherence. The underlying psychometrics were carried out through a reliability, validity and a correlational approach (objective III). These were undertaken in order to have a usable instrument of indebtedness trait in the Spanish-speaking population.

## Materials and Methods

### Participants

Two independent samples were selected to carry out our objectives (I, II, and III). A convenience sampling method was used for both cases. Participants collaborated voluntarily and received no compensation.

First of all, a sample of 229 Spanish undergraduate psychology students (162 women, 70.74%, and 67 men, 29.26%) with an age range between 18 and 45 years, *M* = 21.77, *SD* = 4.11 participated in the exploratory factor analysis, and convergent, discriminant and correlational validity analysis. Secondly, a sample of 200 Spanish undergraduate psychology students participated in the confirmatory factor analysis (145 women, 72.5%, and 55 men, 27.5%). They had an age range of between 18 and 45 years, *M* = 2180, *SD* = 4.45.

### Instruments

The instruments are described according to the aims of the study. First, to test the psychometric properties of the scale under study, the following instruments were selected:

The Revised Indebtedness scale (IS-R, Elster et al., 2005; Van Gelder et al., 2007), which is the scale under study. This was employed in all the steps for the analysis (objective I, II). It was originally developed by Greenberg (1980). This is a 22-item scale. For the adaptation of the scale to Spanish, a back-translation process was performed, as previously recommended in the literature (Muñiz et al., 2013). The final translation completed by the participants appears in the Appendix. The mean corrected item-total correlation was 0.485, Cronbach’s alpha was 0.88, and Spearman-Brown was 0.83. Thus, the revised indebtedness scale (IS-R) had good internal consistency, and might be a useful measure of this construct. Originally, the scale ranged from -3 (strongly disagree) to +3 (strongly agree), but in the current research the participants rate the items from 1 (strongly disagree) to 6 (strongly agree) because this format is more familiar for the Spanish population.

To test the validity, two items were chosen in order to perform convergent validity analyses. The items were the following: Q1: Relationship of Exchange: “Normally, nobody gives anything without expecting something in return from you”; Q2: “Indebtedness and uneasiness when receiving favors; I do not like having favors done for me, because that makes me feel indebted.” The items were answered using a Likert-type scale (strongly disagree) to 7 (strongly agree).

The Gratitude Questionnaire six-item form (GQ-6) (McCullough et al., 2002) was also chosen for exploring the relationship between Gratitude and debt. In this study, the Spanish version used (Bernabé-Valero et al., 2013) is a scale that was adapted from the GQ-6, where item 6 was removed because of empirical and theoretical reasons. It is a 5-item self-report scale that assesses individual differences in the tendency to experience gratitude in daily life. Responses range from 1 to 7 on a 7-point Likert-type scale (1 = strongly disagree and 7 = strongly agree). Possible scores ranged from 5 to 35, with higher scores indicating a higher level of gratitude. The GQ-5 internal consistency ranged from α = 0.77.

Interpersonal Gratitude (GI). This is a subscale of the G-20 Gratitude Questionnaire (Bernabé-Valero et al., 2014). This subscale measures gratitude for interpersonal situations. It has been considered to be the most suitable because it makes explicit the same interpersonal context as the scale of IS-R. This subscale, together with the rest of the scales (gratitude to the suffering, recognition of the gifts and expression of gratitude) seeks to measure gratitude as a construct understood in terms of existential attitude (also considering the distinction between interpersonal and transcendental gratitude). The questionnaire employs a 7-Likert scale that indicated the degree of agreement/disagreement. The scale showed a good internal consistency, α = 0.83.

Also for objective III, the subscale *purpose in life* of the Ryff Scale of Psychological Well-Being (SPWB; Ryff, 1989) was selected for the analysis of discriminant validity. This is a self-reported scale, which assesses psychological well-being from an eudaimonic conception. This measures an individual’s well-being at a particular moment in time within each of 6 dimensions: autonomy, environmental mastery, personal growth, positive relations with others, purpose in life, self-acceptance (Ryff and Singer, 2008). A Spanish 39-item Likert scale (1 = strongly agree and 6 = strongly disagree) was used (Díaz et al., 2006). For each category, a high score indicates that the respondent has a mastery of that area in his or her life. Conversely, a low score shows that the respondent struggles to feel comfortable with that particular concept. The whole SPWB showed high internal consistency α = 0.89, and specifically for the subscale *purpose in life*, the internal consistency was α = 0.83 (Díaz et al., 2006).

### Analysis

The analyses were developed through the SPSS 22 and Amos 18.0 module, following the previous literature (Zenger et al., 2015; Sun and Jiang, 2017). The analyses are divided into the three main objectives which are as follows: (I) The study of psychometric properties of the IS-R scale in the Spanish population, (II) the confirmation factor analysis in the scale under study, and (III) an exploration of potential correlation across other related or unrelated factors. This explores the correlations of the debt with other variables in order to test the validity of the scale and its relationship with other related variables.

### Objective I

In order to examine the adequacy of indebtedness for the Spanish population in terms of psychometric properties, an exploratory factor analysis (EFA) was conducted. Assumptions were checked to ensure the application of factor analysis, such as high sample size, multivariate normality, linearity and correlation between variables (Comrey, 1973; Tabachnick and Fidell, 1989). Moreover, in order to find the suitable number of factors, Cattell’s scree-sediment graph (Cattell, 1966) as well as the eigenvalue (Kaiser, 1960) were used. In this way, the internal consistency of the scale was evaluated through Cronbach’s Alpha, items of homogeneity, KMO index and the Bartlett test of Sphericity (Kaiser, 1974).

### Objective II

After removing the factorial solution, the second step was to proceed to the completion of the confirmatory factor analysis (CFA) though an independent sample, accompanied by the goodness of fit indices. No rotation of the data was employed. Confirmation of the adequacy of the model has been used within the absolute fit indices; the chi-square statistic X^2^ (Jöreskog and Sörbom, 1979; Saris and Stronkhorst, 1984); and its ratio among degrees of freedom where values under 2 are recommendable. In terms of incremental fit indices, the comparative fit index (CFI), was selected. This follows a range of values between 0 and 1 and the reference value is 0.90 (Bollen, 1989; Bentler, 1990; Hu and Bentler, 1998). Finally, within parsimony adjustment indices, the error of the root mean square approximation (RMSEA) was employed. Similarly, the smaller its value, the better the fit, with the reference value being 0.05 (Steiger and Lind, 1980).

### Objective III

The Pearson correlation coefficient in order to examine convergence or divergence with the variables under study was employed. More precisely, when testing the criterion validity of the scale, this was correlated with two items of indebtedness, purpose in life and the gratitude construct.

## Results

This section is divided in terms of objectives, as mentioned in the Analysis section.

### Objective I: Psychometric Properties of the Scale IS-R

#### Internal Consistency

The Cronbach’s alpha on the scale depicted optimal values, with α = 0.919, and the percentage of total variance explained of 49.17%. Table 1 presents the descriptive analysis, homogeneity items, Cronbach’s alpha, kurtosis, skewness, and exploratory factor loadings between items.

**TABLE 1:** Means, standard deviation, item homogeneity, kurtosis, skewness, and exploratory factor loadings for the four factors.

**Item wording**	**Mean**	***SD***	**H^2^**	**Kurtosis**	**Skewness**	**Cronbach’s α**
Factor 1	4.08	1.05	0.41–0.65	−0.32	−0.44	0.81
Factor 2	2.76	1.05	0.44–0.64	−0.47	0.33	0.82
Factor 3	3.51	1.08	0.54–0.80	−0.50	0.06	0.83
Factor 4	2.93	1.15	0.42–0.64	−0.53	0.36	0.75

**SD* = standard deviation; H^2^ = Homogeneity range.*

#### Exploratory Factor Analysis

In relation to the validity of Exploratory Factor Analysis (EFA), the Bartlett’s test of sphericity was *p* < 0.001 with a value of chi-square 2254.78 (df = 231) and the sample index value of Kaiser-Meyer-Olkin (KMO) was 0.93. The scree-test (Cattell, 1966) recommended a four factor solution.

### Objective II: Factor Structure of the IS-R Scale

#### Confirmatory Factor Analysis

The CFA has confirmed through an independent sample (*n* = 200) the existence of four factors. The model presented an optimal fit. The goodness of fit indices global scale was: *χ*^2^ = 363.21, *p* < 0.001 (df = 203), *χ*^2^/df = 1.79, CFI = 0.914, IFI = 0.915, and RMSEA = 0.06.

#### Validity

In terms of qualitative analysis of the factors from the items, it has been pointed out that each factor shows a high coherence in terms of content. Moreover, with regards to the measurement of different facets of the debt appearing on the scale. To summarize the information of each factor, factors were labeled as follows:

Factor 1: Debt for material aspects (Items 3, 5, 6, 7, 8, 9, and 12): in this factor the information related to the discomfort or aversion to contract material debts is described.

Factor 2: Self-sufficiency and discomfort in receiving help (Items 2, 4, 11, 13, 14, and 15): refers to the discomfort of receiving help and favors from others with an attitude of self-sufficiency.

Factor 3: Moral self-demand in the reception of help (Items 1, 10, 18, 19, and 21): a high score in these items indicates that the participants go beyond the rules of exchange; they are self-demanding to be quick in returning the favor. Moreover, the feeling of obligation to return more than the favor received is described here, as well as the return of all favors as indicative of being “a good friend.”

Factor 4: Debt in the receipt of gifts (Items 16, 17, 20, and 22) describes various emotional responses of discomfort, concern and not enjoyment when receiving gifts and relief if they are not received.

### Objective III: Criterion Validity

To test the criterion validity of the scale, this was correlated between the *purpose on life* construct and other theoretical constructs associated with debt. More precisely, with items on the Exchange Relationships (Q1) and the discomfort with the reception of favors (Q2) for the convergent validity. Table 2 depicts the positive correlation of debt with Q1 and Q2. As expected, Indebtedness did not correlate with Purpose in life analyzing discriminant validity.

**TABLE 2:** Pearson coefficients among factors R-Indebtedness, Q1, Q2, and Happiness for construct validity.

	**Q1 (Debt)**	**Q2 (Debt)**	**Purpose**	**Gratitude**	**Gratitude**	**Factor 1**	**Factor 2**	**Factor 3**	**Factor 4**
			**on life**	**(GQ5)**	**interpersonal (GI)**				
Q1 (Debt)	1								
Q2 (Debt)	0.439^∗∗^	1							
Purpose on life	−0.164^*^	−0.136^*^	1						
Gratitude (GQ5)	−0.212^∗∗^	−0.233^∗∗^	0.489^∗∗^	1					
Gratitude (GI)	−0.142^*^	−0.137^*^	0.455^∗∗^	0.479^∗∗^	1				
Factor 1	0.207^∗∗^	0.349^∗∗^	−0.032	−0.112	−0.076	1			
Factor 2	0.340^∗∗^	0.470^∗∗^	−0.113	−0.200^∗∗^	−0.147^*^	0.636^∗∗^	1		
Factor 3	0.260^∗∗^	0.243^∗∗^	−0.050	−0.100	0.019	0.626^∗∗^	0.652^∗∗^	1	
Factor 4	0.264^∗∗^	0.289^∗∗^	−0.155^*^	−0.172^∗∗^	−0.120	0.445^∗∗^	0.572^∗∗^	0.594^∗∗^	1

^*^p < 0.05; ^∗∗^*p* < 0.01.

The relationships between gratitude and indebtedness were as follows: on the one hand, all the indebtedness factors are inversely correlated with gratitude as the GQ5, although only F2 (Self-sufficiency and discomfort in receiving help) and F4 (Debt in the receipt of gifts) are so to a significant extent. However, the Interpersonal Gratitude scale of the G-20 depicted a low correlation with the F2.

## Discussion and Conclusion

The aim of this work was to examine the psychometric properties of the R-Indebtedness Scale to ensure an adaption to the Spanish population. For this reason, a 4-factor solution structure was found with interrelated factors. The factors have a high coherence in their content, so it has been possible to label and describe them.

With regards to the convergent validity, we can affirm that it is high since there are significant, positive correlations between the 4 factors of the R-Indebtedness scale and the Q1 of Exchange Relationships and the Q2 of Discomfort upon the receipt of favors. With regards to discriminant validity, we observed that there is no correlation between *Purpose in life* and all IS-R factors, supporting the idea of good discriminant validity.

The relationships between gratitude and indebtedness are complex: on the one hand, all the indebtedness factors are inversely correlated with gratitude such as the GQ5, although only F2 (Self-sufficiency and discomfort in receiving help) and F4 (Debt in the receipt of gifts) are so to a significant extent. These results are consistent with previous literature in which significant inverse relationships are obtained between gratitude and debt measured as traits (e.g., Elster et al., 2005; Van Gelder et al., 2007; Watkins et al., 2017). However, relations with Interpersonal Gratitude are null, except for F2, which also correlates negatively and significantly, although with a very low value (−0.147). A potential explanation of why F2 and F4 are inversely correlated could be because the content of these factors would be close to what Ting (2017) calls external indebtedness, which implies that in these exchange situations, the beneficiary is unable to evoke feelings of gratitude.

Also, along similar lines, we argue that F1 (Debt for material aspects) and F3 (Moral self-demand in the reception of help) imply less aversion, and therefore, aspects of indebtedness compatible with gratitude are conceivable and they may not necessarily have an inverse relationship. In other words, people who scored high in these two aspects of indebtedness may feel the importance of returning the favor and not wanting to owe material aspects, but this would not always be related to the fact of being able to thank the positive aspects of the reception of the gift. These dispositional aspects of indebtedness would not produce a decrease in the disposition to gratitude, and may be at the expense of other situational factors such as the emergence of the gratitude emotion. Therefore, we propose that there are various degrees of negative emotionality surrounding indebtedness, and that factors 2 and 4 may have a greater degree of discomfort or negative emotionality. On the other hand, the results regarding interpersonal gratitude go further as concerns not obtaining correlations with the debt. We think it is important to use instruments that measure the variables in the same context as the case of IS-R and Interpersonal Gratitude since other instruments may be measuring another type of gratitude and may lead to other results. More research would be required in this area.

On the other hand, these results highlight the importance of having conceptually well-defined instruments with good psychometric properties, such as the IS-R scale that we have analyzed. The fact of being able to count on the 4 labeled factors allows us to carry out different analyses with greater precision. In addition, it is a useful tool to be used in various statistical analyses such as Mediation and SEM since you can use the factors that have a smaller number of indicators than the global scale, allowing the models to adjust better. In the same way, we have been able to benefit from an instrument of measurement that defines the types of gratitude (interpersonal, transcendental and faced with suffering), which has allowed us to relate it to the debt in a more specific interpersonal context.

We believe that this adaptation of the instrument to the Spanish and Spanish-speaking population will allow us to advance in numerous studies that help in the delimitation of debt and gratitude and shed light on the complex dynamics that both constructs manifest.

## Ethics Statement

All subjects gave written informed consent in accordance with the Declaration of Helsinki. The protocol was approved by the Universidad Católica de Valencia committee (number UCV2017-2018-28).

## Author Contributions

GB-V conceived of the presented idea. GB-V, II-M, JB-M and CM-T developed the theory and performed the computations. All authors discussed the results and contributed to the final manuscript.

## Conflict of Interest Statement

The authors declare that the research was conducted in the absence of any commercial or financial relationships that could be construed as a potential conflict of interest.

## Appendix

### Escala De Disposición a La Deuda (Is-R)

Por favor, marque con una cruz el número que mejor representa su acuerdo o desacuerdo en función de la escala que le presentamos. No hay respuestas correctas o incorrectas, solo conteste aquello que se ajuste mejor a sus experiencias.

**TABLE 3:**

**Fuertemente en desacuerdo**					**Fuertemente de acuerdo**
1	2	3	4	5	6

(1) Si un amigo me hiciese un favor, me aseguraría de compensarlo tan rápidamente como fuera posible.
(2) Deber a alguien un favor me hace sentir incómodo.
(3) Yo no pediría dinero prestado a un amigo a menos que fuera absolutamente necesario.
(4) Pedir ayuda a otras personas les da poder sobre tu vida.
(5) Nunca sería un prestatario (deudor) o un prestamista.
(6) Me sentiría avergonzado si alguien me recordase una deuda que tuviese.
(7) Como regla general, no acepto un favor si no puedo devolverlo.
(8) Si alguien me pagara la cena o me invitara a comer a su casa, yo me sentiría obligado a comprar a cenar la próxima vez o invitar a mi casa.

(9) Me sentiría muy molesto si descubriese que me había olvidado de devolver algo que se me había prestado.
(10) Si a alguien le nace ayudarme de alguna manera, me siento, me siento como si yo debiera.
hacer más por ellos que simplemente devolver el favor.
(11) Cuando alguien me hace un favor, a menudo me molesta porque de inmediato me preocupo por cómo lo devolveré.
(12) Me gusta asegurarme de que no le debo nada a nadie.
(13) Me encuentro preocupado sobre si podré pagar todos los favores que he recibido.
(14) Cuando alguien me da algo, o me hace un favor, generalmente siento un poco de incomodidad al principio.
(15) Prefiero hacer las cosas por mí mismo a que alguien me ayude porque no me gustaría sentirme obligado a devolver el favor.
(16) Si no recibo regalos, perfecto.
(17) Si alguien me comprase un regalo caro, me preocuparía mucho si sería capaz de devolverlo.
(18) En las buenas amistades se debe asegurar que se pagan todos los favores que se han recibido de un amigo.
(19) Si alguien me hace un favor, por lo general trato de devolverlo lo más pronto posible.
(20) Me sentiría incómodo si ahora mismo alguien me sorprendiese con un caro y gran regalo.
(21) Ser capaz de devolver un favor o un regalo me proporciona un gran alivio.
(22) Tengo problemas para disfrutar de los regalos que me hacen los demás porque me preocupo acerca de lo que les daré a cambio.

## References

[B1] Algoe S. B. Gable S. L. Maisel N. C. (2010). It’s the little things: everyday gratitude as a booster shot for romantic relationships. *Pers. Relationsh.* 17 217–233. 10.1111/j.1475-6811.2010.01273.x

[B2] Bentler P. M. (1990). Comparative fit indices in structural models. *Psychol. Bull.* 107 238–246. 2320703 10.1037/0033-2909.107.2.238

[B3] Bernabé-Valero G. García-Alandete J. Gallego-Pérez J. F. (2013). Comparative analysis of two models of the gratitude questionnaire-six items form. *Rev. Latinoam. Psicol.* 45 279–288.

[B4] Bernabé-Valero G. García-Alandete J. Gallego-Pérez J. F. (2014). Construcción de un cuestionario para la evaluación de la gratitud: el Cuestionario de Gratitud-20 ítems (G-20). *Anal. Psicol.* 30 278–286.

[B5] Bernabé-Valero G. Moret-Tatay C. Navarro-Sancho T. (2018). Does being a grateful person increase one’s level of well-being? The mediating role of indebtedness. *bioRxiv*

[B6] Bock D. E. Folse J. A. G. Black W. C. (2016). When frontline employee behavior backfires: distinguishing between customer gratitude and indebtedness and their impact on relational behaviors. *J. Serv. Res.* 19 322–336. 10.1177/1094670516633754

[B7] Bollen K. A. (1989). A new incremental fit index for general structural equations models. *Soc. Methods Res.* 17 303–316. 10.1177/0049124189017003004

[B8] Cattell R. B. (1966). The scree test for the number of factors. *Multivariate Behav. Res.* 1 245–276. 10.1207/s15327906mbr0102_10 26828106

[B9] Comrey A. L. (1973). *A First Course in Factor Analysis.* New York, NY: Academic Press.

[B10] Díaz D. Rodríguez-Carvajal R. Blanco A. Moreno-Jiménez B. Gallardo I. Valle C. (2006). *Adaptación Española de Las Escalas de Bienestar Psicológico de Ryff.* Available at: http://www.redalyc.org/articulo.oa?id=72718337 (accessed May 10, 2017). 17296089

[B11] Elster B. Maleki L. McLeod L. Watkins P. (2005). How are gratitude and indebtedness related?. *Paper Presentation to the 85th Annual Convention of the Western Psychological Association*, Portland, OR.

[B12] Fredrickson B. L. (2004). “Gratitude, like other positive emotions, broadens and builds,” in *The Psychology of Gratitude*, eds Emmons R. A. McCullough M. E. (New York, NY: Oxford University Press), 145–166.

[B13] Goei R. Boster F. J. (2005). The roles of obligation and gratitude in explaining the effect of favors on compliance. *Commun. Monogr.* 72 284–300. 10.1080/03637750500206524

[B14] Gordon A. M. Impett E. A. Kogan A. Oveis C. Keltner D. (2012). To have and to hold: gratitude promotes relationship maintenance in intimate bonds. *J. Pers. Soc. Psychol.* 103:257. 10.1037/a0028723 22642482

[B15] Gray S. A. Emmons R. A. Morrison A. (2001). “Distinguishing gratitude from indebtedness in affect and action tendencies,” in *Póster Presentado en el Encuentro Anual de la American Psychological Association*, (San Francisco, CA: EEUU).

[B16] Greenberg M. S. (1980). “A theory of indebtedness,” in *Social Exchange*, eds Gergen K. J. Greenberg M. S. Willis R. H. (Boston, MA: Springer), 3–26. 10.1007/978-1-4613-3087-5_1

[B17] Hu L. T. Bentler P. M. (1998). Fit indices in covariance structure modeling: sensitivity to underparameterized model misspecification. *Psychol. Methods* 3 424–453. 10.1037//1082-989x.3.4.424

[B18] Jöreskog K. G. Sörbom D. (1979). *Advanced in Factor Analysis and Structural Equation Models.* Cambridgem, MA: ABT.

[B19] Kaiser H. F. (1960). The application of electronic computers to factor analysis. *Educ. Psychol. Meas.* 20 141–151. 10.1177/001316446002000116

[B20] Kaiser H. F. (1974). An index of factorial simplicity. *Psychometrika* 35 401–415.

[B21] Kotani M. (2002). Expressing gratitude and indebtedness: Japanese speakers’ use of” I’m sorry” in english conversation. *Res. Lang. Soc. Interact.* 35 39–72. 10.1207/s15327973rlsi35-1_2

[B22] Lazarus R. S. Lazarus B. N. (1994). *Passion and Reason: Making Sense of Our Emotions.* New York, NY: Oxford University Press.

[B23] Mathews M. A. Green J. D. (2010). Looking at me, appreciating you: self-focused attention distinguishes between gratitude and indebtedness. *Cogn. Emot.* 24 710–718. 10.1080/02699930802650796

[B24] Mathews M. A. Shook N. J. (2013). Promoting or preventing thanks: regulatory focus and its effect on gratitude and indebtedness. *J. Res. Pers.* 47 191–195. 10.1016/j.jrp.2013.01.001

[B25] Mayer J. D. Salovey P. Gombergkaufman S. Blainey K. (1991). A broader conception of mood experience. *J. Pers. Soc. Psychol.* 60 100–111. 10.1037//0022-3514.60.1.100 1995832

[B26] McCullough M. E. Emmons R. A. Tsang J. A. (2002). The grateful disposition: a conceptual and empirical topography. *J. Pers. Soc. Psychol.* 82:112. 10.1037//0022-3514.82.1.112 11811629

[B27] Muñiz J. Elosua P. Hambleton R. K. (2013). Directrices para la traducción y adaptación de los tests: segunda edición. *Psicothema* 25 151–157. 23628527 10.7334/psicothema2013.24

[B28] Naito T. Sakata Y. (2010). Gratitude, indebtedness, and regret on receiving a friend’s favor in Japan. *Psychologia* 53 179–194. 10.2117/psysoc.2010.179

[B29] Naito T. Wangwan J. Tani M. (2005). Gratitude in university students in Japan and Thailand. *J. Cross Cult. Psychol.* 36 247–263. 10.1177/0022022104272904

[B30] Oishi S. Koo M. Lim N. Suh E. M. (2019). When gratitude evokes indebtedness. *Appl. Psychol. Health Well Being* 10.1111/aphw.12155 [Epub ahead of print] 30724033

[B31] Peng C. Nelissen R. M. Zeelenberg M. (2018). Reconsidering the roles of gratitude and indebtedness in social exchange. *Cogn. Emot.* 32 760–772. 10.1080/02699931.2017.1353484 28718342

[B32] Ryff C. D. (1989). Beyond ponce de leon and life satisfaction: new directions in quest of successful ageing. *Int. J. Behav. Dev.* 12 35–55. 10.1177/016502548901200102

[B33] Ryff C. D. Singer B. H. (2008). Know thyself and become what you are: a eudaimonic approach to psychological well-being. *J. Happiness Stud.* 9 13–39. 10.1007/s10902-006-9019-0

[B34] Saris W. E. Stronkhorst H. (1984). *Casual Modeling in Non-Experimental Research: An Introduction to the LISREL Approach.* Amsterdam: Sociometric Research Foundation.

[B35] Setti I. van der Velden P. G. Sommovigo V. Ferretti M. S. Giorgi G. O’Shea D. (2018). Well-being and functioning at work following thefts and robberies: a comparative study. *Front. Psychol.* 9:168. 10.3389/fpsyg.2018.00168 29515488 PMC5826257

[B36] Solom R. Watkins P. C. McCurrach D. Scheibe D. (2016). Thieves of thankfulness: traits that inhibit gratitude. *J. Posit. Psychol.* 12 120–129. 10.1080/17439760.2016.1163408

[B37] Steiger J. H. Lind C. (1980). Statistically based tests for the number of common factors. *Paper Presented at the Annual Meeting of the Psychometric Society*, Iowa City, IA.

[B38] Sun P. Jiang H. (2017). Psychometric properties of the Chinese version of core self-evaluations scale. *Curr. Psychol.* 36 297–303. 10.1007/s12144-016-9418-2

[B39] Tabachnick B. G. Fidell L. S. (1989). *Using Multivariate Statistics*, 2nd Edn Northridge, CA: Harper Collins Publishers.

[B40] Ting S. C. (2017). The difference between gratitude and indebtedness. *Am. Int. J. Contemp. Res.* 7 55–62.

[B41] Tsang J. (2006a). Gratitude and prosocial behaviour: an experimental test of gratitude. *Cogn. Emot.* 20 138–148. 10.1080/02699930500172341 25300047

[B42] Tsang J. (2006b). The effects of helper intention on gratitude and indebtedness. *Motiv. Emot.* 30:198204.

[B43] Tsang J. A. (2007). Gratitude for small and large favors: a behavioral test. *J. Posit. Psychol.* 2 157–167. 10.1080/17439760701229019

[B44] Van Gelder M. Ruge L. Brown A. Watkins P. C. (2007). Gratitude and indebtedness are distinct traits: differential associations with well-being. *Paper Presentation to the Annual Convention of the Western Psychological Association*, Portland, OR.

[B45] Washizu N. Naito T. (2015). The emotions sumanai, gratitude, and indebtedness, and their relations to interpersonal orientation and psychological well-being among Japanese university students. *Int. Perspect. Psychol. Res. Pract. Consult.* 4:209 10.1037/ipp0000037

[B46] Watkins P. Scheer J. Ovnicek M. Kolts R. (2006). The debt of gratitude: dissociating gratitude and indebtedness. *Cogn. Emot.* 20 217–241. 10.1080/02699930500172291

[B47] Watkins P. C. (2014). “What inhibits gratitude?,” in *Gratitude and the Good Life*, (Berlin: Springer), 213–223. 10.1007/978-94-007-7253-3_12

[B48] Watkins P. C. Bell J. D. McLaughlin T. Dan Scheibe (2017). “Gender, gratitude, and indebtedness: the debt of gratitude is heavier for men than women,” in *Poster Presentation to the Annual Convention of the Association for Psychological Science*, Boston, MA.

[B49] Watkins P. C. Elster B. N. Maleki L. McLeod L. C. (2005). How are gratitude and indebtedness related? *Paper Presentation at the 2005 Annual Convention of the Western Psychological Association*, Portland, OR.

[B50] Watkins P. C. McLaughlin T. Parker J. P. (2019). “Gratitude and subjective well-being: cultivating gratitude for a harvest of happiness,” in *Scientific Concepts Behind Happiness, Kindness, and Empathy in Contemporary Society*, ed. Silton N. R. (Pennsylvania, PA: IGI Global), 20–42. 10.4018/978-1-5225-5918-4.ch002

[B51] Zenger M. Körner A. Maier G. W. Hinz A. StöbelRichter Y. Brähler E. (2015). The core self-evaluation scale: psychometric properties of the german version in a representative sample. *J. Pers. Assess.* 97 310–318. 10.1080/00223891.2014.989367 25531806

